# Granulocyte colony-stimulating factor (GCSF) fused with Fc Domain produced from *E. coli* is less effective than Polyethylene Glycol-conjugated GCSF

**DOI:** 10.1038/s41598-017-06726-7

**Published:** 2017-07-25

**Authors:** Bich Hang Do, Hyo Jeong Kang, Jung-A Song, Minh Tan Nguyen, Sangsu Park, Jiwon Yoo, Anh Ngoc Nguyen, Grace G. Kwon, Jaepyeong Jang, Mihee Jang, Sunju Lee, Seoungjun So, Seongrak Sim, Kyung Jin Lee, Mark J. Osborn, Han Choe

**Affiliations:** 1 Department of Physiology, Asan-Minnesota Institute for Innovating Transplantation, Bio-Medical Institute of Technology, University of Ulsan College of Medicine, Asan Medical Center, Seoul, 05505 Korea; 2 Department of Convergence Medicine, Asan Institute for Life Sciences, University of Ulsan College of Medicine, Asan Medical Center, Seoul, 05505 Korea; 3 Department of Pediatrics, Division of Blood and Marrow Transplantation, Center for Genome Engineering, Stem Cell Institute, University of Minnesota, Minneapolis, MN 55455 USA

## Abstract

Human granulocyte colony-stimulating factor (GCSF) is a well-known cytokine for neutropenia treatment. However, daily injections are required due to the short circulating half-life of the protein. To overcome this bottleneck, we fused GCSF with the Fc domain of IgG1 at the C terminus (GCSF-Fc) and with the maltose binding protein (MBP) tag at the N-terminus and expressed it as a soluble protein in the cytoplasm of *E. coli*. We also conjugated PEG aldehyde to GCSF to make PEG-GCSF. The bioactivities of GCSF-Fc and PEG-GCSF were similar to native GCSF using the mouse M-NFS-60 myelogenous leukemia cell line. The EC_50_ dose-response curves for GCSF, GCSF-Fc and PEG-GCSF were 37 ± 12 pM, 75 ± 13.5 pM and 46 ± 5.5 pM, respectively. When the proteins were injected into neutropenic rats, the group injected with PEG-GCSF showed the highest and fastest recovery of neutrophils, followed by GCSF-Fc and GCSF. ELISA assay revealed the PEG-GCSF had the longest plasma circulation (>72 h), followed by GCSF-Fc (>48 h) and GCSF (~24 h), which is consistent with the *in vivo* activities of the proteins. In summary, the GCSF-Fc purified from *E. coli* was not as efficient as PEG-GCSF in treating neutropenic rats.

## Introduction

Human granulocyte colony-stimulating factor (GCSF) is a 19 kDa cytokine that is approved by the US FDA for the treatment of neutropenia patients due to its ability to control the production, differentiation and function of granulocytes^1, 2^. Although GCSF is an effective treatment for the patients, the protein has a short circulating half-life, 3.5–3.8 h^3^, which necessitates daily injections and is fiscally burdensome. To prolong the *in vivo* half-life of the GCSF, it has been conjugated with polyethylene glycol (PEG)^3–5^, human serum albumin^6, 7^, or fused with the Fc domain of IgG^8, 9^.

PEG is a polymer of ethylene oxide with some unique physicochemical properties. PEG has both hydrophobicity and hydrophilicity and tends to occupy a large volume in an aqueous environment by the chain flexibility and extensive hydration. Also, it shows inertness and acceptable toxicological characteristics^10^. As such, the covalent modification of proteins with PEGs has become a popular strategy in the biopharmaceutical industry to increase the serum half-life and reduce immunogenicity^11–13^. The PEG-GCSF has been shown to extend the circulating half-life up to 42 hours, which allows effective administration once per chemotherapy cycle^5^.

Generally, IgGs have a long circulating half-life (14–21 days) and the persistence of IgG involves the “protective” neonatal Fc receptor (FcRn). IgGs bind to the FcRn in acidic lysosomal compartments and are recycled back into the bloodstream at physiological pH^14^. Consequently, the Fc region of IgG has been applied to extend the half-life of many target proteins. An example of this is Etanercept that is a fusion of the TNF receptor and Fc region that is approved for autoimmune disease^15^.

Previous studies showed the fusion of GCSF/IgG1-Fc which was produced in mammalian cells has a 5- to 8-fold longer half-life than that of GCSF^8, 9^. In this study, we conjugated 20 kDa PEG to the N-terminus of purified GCSF by reductive alkylation method at pH 6. We also generated a fusion of the IgG1 Fc domain to the C-terminus of GCSF and purified the chimeric protein. To evaluate the *in vitro* bioactivity of PEG-GCSF and GCSF-Fc, the candidate proteins were cultured with mouse myeloblast M-NFS-60 cells. In addition, the pharmacokinetic and granulocytic recovery capabilities of GCSF, PEG-GCSF and GCSF-Fc were also compared in a neutropenic rat model.

## Materials and Methods

### Materials

Shuffle T7 Express cell was obtained from New England Biolabs (Ipswich, MA, USA). Isopropyl β-D-1-thiogalactopyranoside (IPTG) was purchased from Anaspec (Fremont, CA). Overlap cloner^TM^ DNA cloning kit and LR recombination enzyme were from Elpis-biotech (Daejeon, Korea). Protein A resin was obtained from Amicogen (Jinju, Korea). Superdex 200 26/60 gel filtration column, HiTrap SP HP cation exchange column, HisTrap HP column were purchased from GE healthcare (Piscataway, NJ). Protein-pak 300SW SEC. 7.5 × 300 mm column was from Waters Corporation (Milford, MA). Dialysis membranes were from Viskase (Darien, IL). Amicon Ultra was from Merck Millipore (Darmstadt, Germany). Limulus Amebocyte Lysate (LAL) assay kit was from Lonza (Basel, Switzerland). Trypsin was obtained from Promega (Madison, WI) and the Zorbax 300SB-C18 column was purchased from Agilent Technology (Waldbronn, Germany). Twenty kDa methoxy polyethylene glycol aldehyde (mPEG-CHO) was from Nanocs (New York, NY). RPMI 1640, fetal bovine serum (FBS), β-mercaptoethanol, penicillin and streptomycin were purchased from Gibco (Grand Island, NY). 3-(4,5-Dimethylthiazol-2-yl)-2,5-diphenyltetrazolium bromide (MTT) was from Amresco (Solon, OH). Cyclophosphamide monohydrate (CPA) and dimethyl sulfoxide (DMSO) were obtained from Sigma (St. Louis, MO). GCSF ELISA kit was from R&D system (Minneapolis, MN). Sprague-Dawley rats were obtained from Orient Bio (Seongnam, Korea).

### Construction of GCSF-Fc plasmid and expression test

One hundred seventy-five amino acids of the human GCSF was optimized for the expression in *E. coli* ^16^. IgG1 Fc domain was inserted into pDest-HMGWA destination vector^17^ at the position right after attB2 sequences using Overlap cloner^TM^ DNA cloning kit. Then, optimized GCSF gene was subcloned into created vector by LR recombination cloning. A tobacco etch virus (TEV) protease recognition site (TEVrs; ENLYFQ^V^G) was inserted between the tag and GCSF. The sequences of the clones were confirmed by DNA sequencing (Macrogen, Daejeon, Korea).

The expression plasmids were transformed into *E. coli* Shuffle T7 Express for expression. The transformed cells were grown in Luria Bertani (LB) broth containing 50 µg/mL ampicillin at 37°C in 200 rpm shaking incubator. When OD_600_ reached around 0.5, 0.5 mM isopropyl-b-D-thiogalactoside (IPTG) was added and cells were continued to incubate at different temperatures and time, 37°C for 3 h, 30°C for 5 h, 25°C for 8 h and 18°C for 16 h to induce protein expression.

### Purification of GCSF-Fc

The culture was scaled up to 1 liter with supplementary IPTG (0.5 mM) at 30°C for 5 h. The collected cells were resuspended into 100 mL buffer containing 20 mM Tris, 5% glycerol (v/v), pH 8. The cells were homogenized by sonication using ultrasonic cell disruptor JY99-IIDN (Ningbo Scientz Biotechnology, Guangdong, China) on ice at 1,000 W for 40 cycles for 10 seconds, followed by intervals of 50 seconds for cooling. After homogenization, the supernatant was collected by centrifugation for 20 min at 27,000 g. Five mL equilibrated protein A resin and TEV protease were added into supernatant. The whole solution was incubated at 4°C for 16 h with gently shaking. The GCSF-Fc was eluted by 0.1 M Glycine pH 3.5 and 1 M Tris pH 9.5 was used to neutralize the eluate. The GCSF-Fc dimer was separated from higher multimers by Superdex 200 26/60 gel filtration column in Tris buffer containing 200 mM NaCl. Based on the chromatogram, the collected GCSF-Fc was analyzed by 10% SDS-PAGE. The endotoxin in final products was removed by Triton^TM^ X-114 and measured by LAL assay.

### Mass analysis of GCSF-Fc

The half of the protein was reduced with 10 mM DTT for 30 min at 60 °C and alkylated with 55 mM IAA for 30 min in the dark, following by digested with trypsin. The digested peptides were resuspended in 0.1% TFA and loaded onto Zorbax 300SB-C18 75 μm i.d. × 15 cm column via a trap column (Zorbax 300SB-C18 300 μm i.d. × 5 mm column). Peptides were then separated in an acetonitrile gradient (buffer A – 0.1% formic acid; buffer B – 100% acetonitrile and 0.1% formic acid) at a flow rate of 200 nl/min with an Agilent 1100 nanoHPLC system (Agilent, USA) and applied on-line to an Q Star XL mass spectrometer (AB Sciex, USA). The gradient was increased from 5% to 40% solution B over 110 min, followed by an increase to 95% B over 1 min, and then 95% B isocratic for 15 min. MS spectra were collected in full scan mode (350–1400 Da) followed by three MS/MS scans of the most intense ions.

### Plasmid construction and purification of GCSF from PDIb´a´-GCSF that used enterokinase protease for tag removal

The expression vector containing PDIb´a´-GCSF with enterokinase recognition sites (EKrs), DDDDK between tag and target was constructed following the protocol of previous publication^16^. Then the expression vector was expressed inside *E. coli* BL21 under the supplementary of 0.5 mM IPTG at 18°C for 16 h.

This GCSF was purified following the preceding report^16^, except using enterokinase protease light chain (EKL) instead of TEV protease to separate tag from GCSF. The EKL was produced using *E. coli* by our laboratory (manuscript in preparation). The purity of purified protein was confirmed on SDS-PAGE.

### Conjugation and purification of PEG-GCSF

Twenty mg recombinant human GCSF was prepared following the protocol of the previous report^16^. Twenty kDa PEG was conjugated to N-terminus of GCSF by the reductive alkylation method at low pH^18^. Briefly, the buffer of 4 mL GCSF at concentration of 5 mg/mL was changed into 0.1 M sodium phosphate buffer pH 6.0 by dialysis. This solution was added to a vial containing 100 mg 20 kDa mPEG-CHO. When PEG was dissolved completely, 82 µL of 1 M sodium cyanoborohydride was added to the reaction mixture. The reaction mixture was gently stirred in dark at 4°C for 16 h. Then sample was diluted 20 times by 20 mM sodium phosphate buffer pH 4.0 and loaded onto 5 mL HiTrap SP HP cation exchange column pre-equilibrated with the same buffer. After washing with 10 column-volume of the same buffer, a linear gradient elution from 0 to 1 M NaCl of the sodium phosphate buffer pH 4 was applied to elute PEG-GCSF. The fractions were analyzed by SDS-PAGE.

### Cell proliferation assay

The M-NFS-60 cells were grown in RPMI-1640 medium containing 10% fetal bovine serum, 1X penicillin and streptomycin, and 0.05 mM β-mercaptoethanol at 37°C in a humidified atmosphere containing 5% CO_2_. The cells (5 × 10^3^ cells/well) were seeded into 96-well plate containing growth medium. Simultaneously, different concentrations of GCSF, PEG-GCSF, and GCSF-Fc (0.001, 0.01, 0.1, 1, 10, 100 ng/mL) were added to each well in a final volume of 100 µL. Phosphate buffered saline (PBS) was used as control sample. After 72 h of incubation, 15 µL of 5 mg/mL MTT was added to each well and the cells were kept incubating at 37°C for further 4 h in dark. After draining the solutions, 100 µL of DMSO was added to each well to completely solubilize the formed aggregates. The optical density of the solution was measured at 570 nm using an ELISA reader.

Protein dose-response proliferation was analyzed using following equation and Microsoft Excel software.

1 $$Re=Bl+(Max-Bl)/(1+{(E{C}_{50}/conc)}^{Hs})$$

The abbreviation is as follows: *Re*, response of the cells; *Bl*, baseline at low concentration; *Max*, the maximum response; *conc*, concentration of the protein; and *Hs*, Hill coefficient of stimulation.

### *In vivo* experiment on neutropenic rats

The research protocol was approved by the Institutional Animal Care and Use Committee of the Asan Institute for Life Science, and mice were maintained in accordance with the Institutional Animal Care and Use Committee guidelines of the Asan Institute for Life Science. All experiments were performed in accordance with relevant guidelines and regulations. Seven weeks 220 g Sprague-Dawley rats were acclimatised for at least 7 days before experiments. Rats were randomly separated into 7 groups with 5 rats in each group. Group 1 was used as control group. Six other groups received an intraperitoneal injection of 100 mg/kg of CPA on day 0 to induce neutropenia. On day 1, these 6 groups received subcutaneous injections of phosphate buffered saline (PBS) (group 2), 100 µg/kg GCSF (group 3), 100 µg/kg PEG-GCSF (group 4), 50 µg/kg GCSF-Fc (group 5), 100 µg/kg GCSF-Fc (group 6) and 300 µg/kg (group 7). More than 3 mL of blood were collected from day 0 to day 12 with heparinized syringe. 200 µl sample from the collected blood were analysed for complete blood count (CBC) using ADVIA 2120i Hematology System (Siemens Healthineers, Erlangen, Germany). The remaining bloods were centrifuged at 1,500 g for 15 min to collect plasma for ELISA assay. The GCSF serum levels on days 2, 3, 4 from group 3, 4 and 7 were quantitated using Quantikine human GCSF ELISA kits.

### Statistical analysis

All data are presented as the mean ± standard error (SE) of n ≥ 3 of 2 independent experiments. Statistical analyses were performed using the SPSS statistical software program (SPSS, version 18.0, Chicago, IL). A Student’s t-test was used to determine the statistical significance of group means. All tests were two-sided and *p*-values less than 0.05 were considered statistically significant.

## Results

### Expression of MBP-GCSF-Fc in *E. coli* and its purification

**Figure 1:**
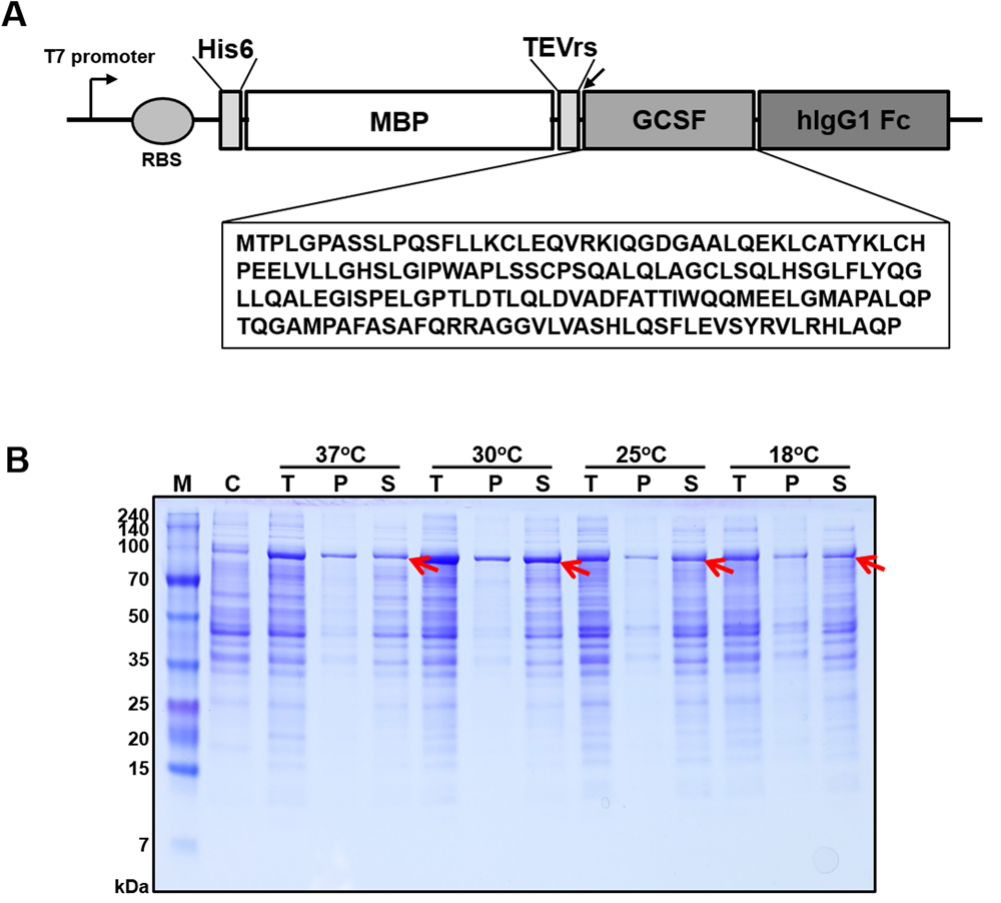
Schematic representation of MBP fused GCSF-Fc construct (**A**) and expression of MBP fused GCSF-Fc at different temperatures (**B**). GCSF gene was fused with a C-terminal IgG1 Fc domain and an MBP tag at the N-terminus. The expression was controlled by the T7 promoter. Arrow between GCSF and MBP indicates TEV cleavage site. His6 is N-terminal to the MBP tag. MBP-GCSF-Fc expression was induced by 0.5 mM IPTG at: 18°C, 25°C, 30°C or 37°C. Arrows indicate the target fusion proteins. The abbreviations are as follows: RBS, ribosome binding site, M, molecular weight marker; C, total cell protein before IPTG induction as a negative control; I, total cell protein after IPTG induction; P, pellet fraction after cell homogenization; S, soluble fraction after cell homogenization.

The vector shown in Fig. 1A containing GCSF with an MBP tag at the N-terminus and IgG1 Fc domain at the C-terminus was generated for use. The expression kinetics of MBP-GCSF-Fc and PDIb´a´-GCSF-Fc in *E. coli* strain BL21 with 0.5 mM IPTG at 18°C for 16 h is shown in Supplementary Fig. S1. During the isolation process the GCSF-Fc after TEV cleavage formed multimers and aggregates hampering further purification (data not shown). Therefore, MBP-GCSF-Fc and PDIb´a´-GCSF-Fc were expressed in Shuffle T7 Express. In our previous study, MBP-GCSF or PDIb´a´-GCSF in BL21(DE3) was expressed well and highly soluble when incubated with 1 mM IPTG at 18°C for 16 h^16^. However, under the conditions of the present study, very few PDIb´a´-GCSF-Fc were expressed (data not shown) and MBP-GCSF-Fc was expressed in low amounts (Fig. 1B). To increase the expression level, several conditions of induction temperatures, 18°C, 25°C, 30°C and 37°C were tested for the expression of MBP-GCSF-Fc. As a result, the expression of protein at 30°C for 5 hrs showed the highest expression and solubility efficiency which were approximately 26.7% and 74.5% respectively (Fig. 1B).

**Figure 2:**
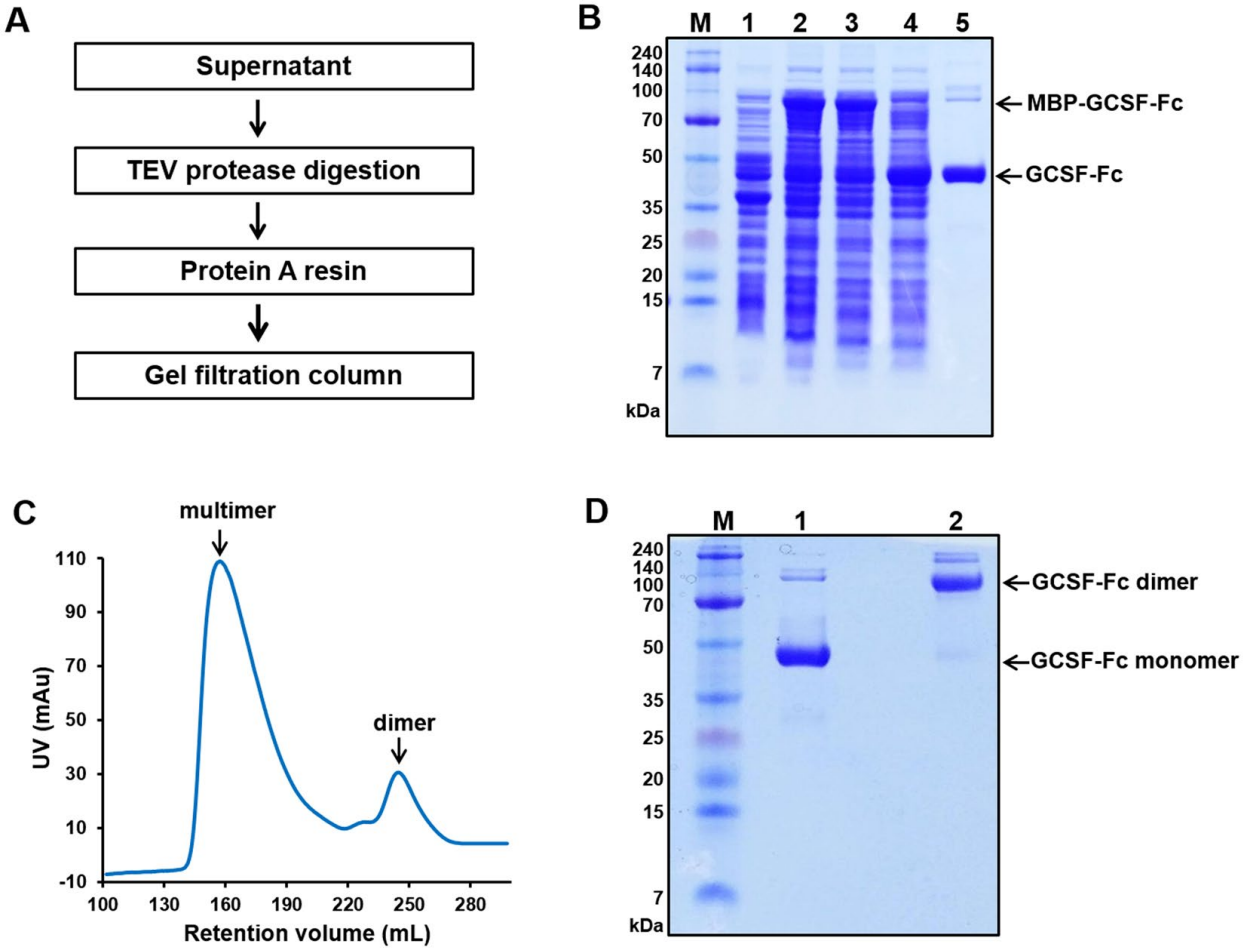
Purification of GCSF-Fc from *E*. *coli*. (**A**) Schematic overview of the GCSF-Fc purification process using protein A resin and gel filtration chromatography. (**B**) SDS-PAGE (10% Tris-tricine gel) analysis of GCSF-Fc through different purification steps. M, molecular weight marker; lane 1, total cell extract before IPTG induction; lane 2, total cell extract after IPTG induction; lane 3, soluble fraction after cell homogenization; lane 4, MBP-GCSF-Fc fusion protein was cleaved by TEV protease; lane 5, final GCSF-Fc after purifying by protein A resin and gel filtration column. The arrows indicate positions of fusion MBP-GCSF-Fc (87.8 kDa) and GCSF-Fc (43.8 kDa). (**C**) Chromatogram of superdex 200 26/60 gel filtration column after protein A resin step to separate homodimers from multimers. GCSF-Fc dimers were collected from 235 mL to 270 mL of retention volume. (**D**) SDS-PAGE of GCSF-Fc under reducing and non-reducing condition. Lane 1, reducing GCSF-Fc (43.8 kDa), lane 2, non-reducing GCSF-Fc shows the homodimer with the size of 87.6 kDa.

After sonication, MBP-GCSF-Fc was present in the supernatant fraction because this protein was soluble after induction with IPTG. When the fusion protein was mixed with TEV protease and protein A resin beads simultaneously, GCSF-Fc was separated from the fusion protein and bound protein A resin. This target protein was eluted off of the beads by low pH buffer (0.1 M Glycine buffer pH 3.5). To prevent the aggregation of protein at this low pH, 1 M Tris buffer pH 9.5 with at a ratio of 1:10 was used to neutralize the eluate. Despite this, some proteins formed higher multimers and to remove these multimers, a gel filtration column was applied. As a result, approximately 90% of GCSF-Fc multimer was removed from GCSF-Fc dimers (Fig. 2C and D). Based on SDS-PAGE analysis, the GCSF-Fc dimer was highly pure (more than 95%) and endotoxin level in the product was less than 1 EU/µg after endotoxin removal by Triton^TM^ X-114. Finally, approximately 1 mg GCSF-Fc was obtained from 1 L of *E. coli* cultured cells.

To confirm the identity of the purified protein and the disulfide bondings, the peptide was subjected to reducing (with DTT) and non-reducing (without DTT) conditions and then treated with trypsin and then analyzed by LC-MS/MS. The m/z peak lists of reducing and non-reducing samples were entered into the MASCOT search database. The result matched to the GCSF and Fc of gamma 3 chain in both reducing and non-reducing condition (Supplementary Fig. S2). Additionally, the m/z value of 780.51^7+^ was observed in non-reducing condition only, confirming two inter-disulfide bonds of C_197_-C_197_ and C_200_-C_200_ in Fc region (Supplementary Figs S3 and S4).

### PEGylation for GCSF

**Figure 3:**
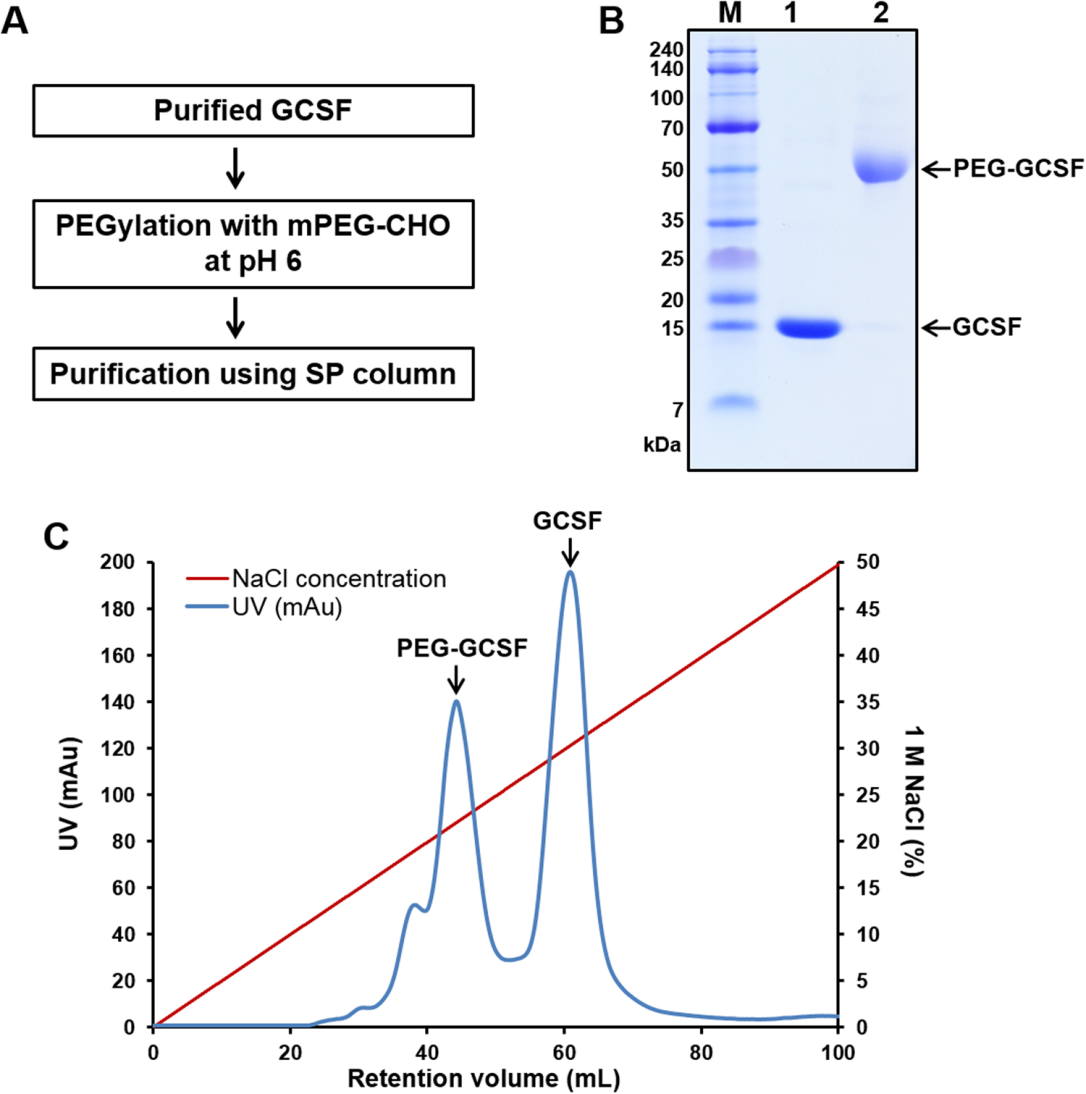
PEGylation process and purification of PEG-GCSF. (**A**) Schematic overview of PEGylation for GCSF and purification of PEG-GCSF. (**B**) SDS-PAGE analysis of GCSF and PEG-GCSF showed monoPEG was conjugated to 1 GCSF molecule. M, molecular weight marker; lane 1, GCSF before PEGylation (18.8 kDa), lane 2, PEG-GCSF after PEGylation step with mPEG-CHO and purification through SP chromatography (38.8 kDa). (**C**) Chromatogram of HiTrap SP HP column to obtain pure PEG-GCSF. PEG-GCSF with higher negative charge was eluted before GCSF.

PEG was conjugated to the N-terminus of GCSF using a reductive alkylation method with 20 kDa mPEG aldehyde at low pH. When the pH was lowered from 8 to 5, GCSF became unstable and formed aggregates (data not shown). To overcome this problem, we lowered the reaction pH to 6 and the protein was stable. After incubation of GCSF with mPEG-CHO and sodium cyanoborohydride, approximately 50% of PEG was conjugated to GCSF (Fig. 3C). The PEG-GCSF was then separated from non-reacted GCSF and mPEG-CHO by a cation exchange column using a sodium phosphate buffer of pH 4, suggesting that the PEGylation increased the stability of GCSF at low pH. Two major peaks were observed (Fig. 3C), and SDS-PAGE analysis of the elution peaks showed that the earlier peak had a single band of approximately 50 kDa that corresponds to PEG-GCSF (Fig. 3B). The non-reacted mPEG-CHO did not bind to the column. Overall, 8 mg of PEG-GCSF was obtained from a starting yield of 20 mg GCSF.

Since the PEGylation efficiency was as low as 50% and the GCSF has one remaining N-terminal glycine residue after TEV cleavage, we purified non-Gly GCSF from PDIb´a´-GCSF cleaved by enterokinase light chain (EKL) protease (Supplementary Fig. S5). This GCSF isolate was subsequently conjugated with mPEG-CHO using the same method as detailed above. As a result, more than 90% of GCSF was conjugated to PEG based on size exclusion chromatography (SEC)-HPLC (Supplementary Fig. S6).

### *In vitro* activity of proteins

**Figure 4:**
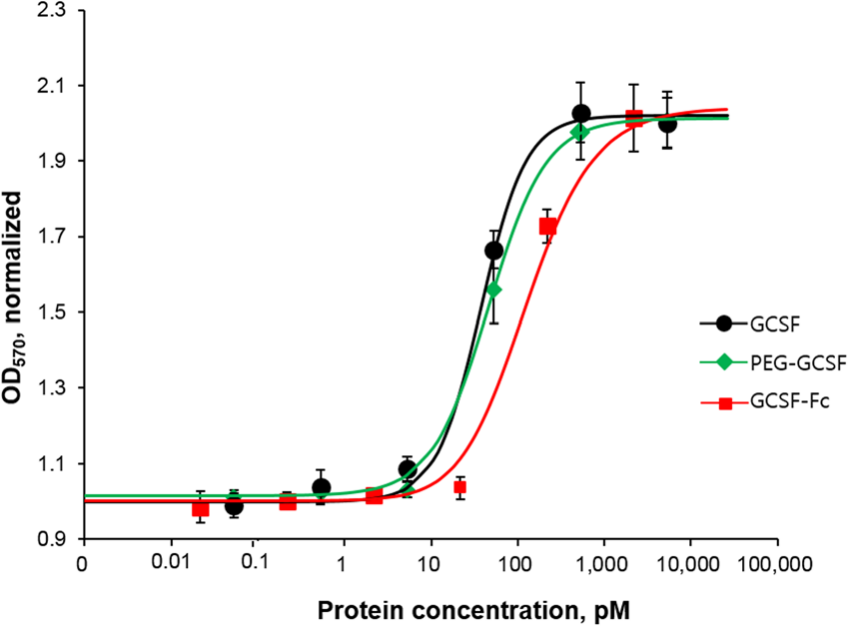
Dose-response curves of purified GCSF-Fc, PEG-GCSF and GCSF on M-NFS-60 cell line. The number of cells was measured at OD_570_ following a standard MTT assay protocol. Data are represented as the mean ± SE of n ≥ 3 of 2 independent experiments.

The biological activity of the purified GCSF-Fc and PEG-GCSF was measured using a proliferation assay with the mouse myelogenous leukemia M-NFS-60 cell line. After incubation of cells with purified GCSF, GCSF-Fc and PEG-GCSF at different concentration for 3 days, the cell number and viability was measured using an MTT assay. These data showed that the number of cells increased dramatically after incubation with all three proteins and followed a sigmoidal dose-response curves (Fig. 4). The EC_50_ of GCSF, GCSF-Fc and PEG-GCSF were highly similar with a range of: 37 ± 12 pM, 75 ± 13.5 pM and 46 ± 5.5 pM, respectively. The hill coefficient was approximately 1.5 for all cases, suggesting that fusion with Fc region or conjugation with PEG did not affect the biological activity of GCSF (Fig. 4).

### *In vivo* bioactivity of proteins

**Figure 5:**
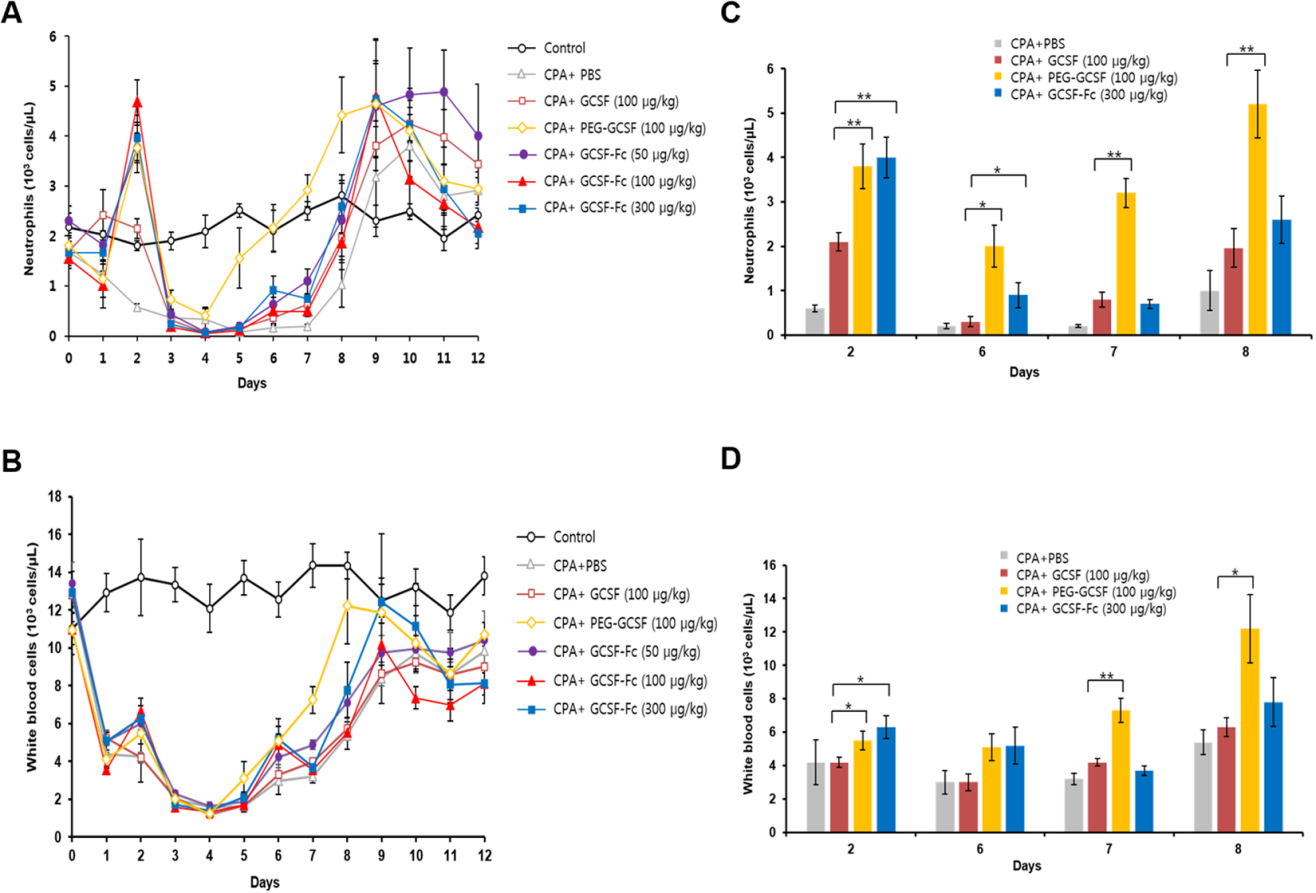
Neutrophils (**A** and **C**) and white blood cells (**B** and **D**) counts in neutropenic rats received single injection of GCSF-Fc, PEG-GCSF and GCSF. On day 0, 5 rats in each group but the control group received 100 mg/kg CPA to induce neutropenia. On day 1, neutropenic groups received injections of PBS, GCSF (100 µg/kg), PEG-GCSF (100 µg/kg), or GCSF-Fc (50 µg/kg, 100 µg/kg and 300 µg/kg). Blood samples from the rat groups over 12 days were sent for complete blood count (CBC) analysis. Data are means ± SE of 5 rats/group. Statistical significance compared to GCSF treatment group: *p < 0.05, **p < 0.01.

To evaluate the effects of the proteins in an *in vivo* setting, we injected rats with 100 mg/kg of cyclophosphamide (CPA) reagent on day 0 with follow on GCSF candidate protein injections on day 1. After CPA application, the level of neutrophils and total white blood cells (WBC) in the peripheral blood was sharply reduced and then began to rise after GCSF protein infusion (day 2) (Fig. 5A and B). On day 2, the amounts of neutrophils and WBCs in rats injected with PEG-GCSF and GCSF-Fc were significantly higher than those in GCSF or the PBS treated group (Fig. 5C and D). Animals treated with PEG-GCSF showed the quickest recovery, followed by GCSF-Fc and GCSF, respectively. Of note, on day 6, neutrophils and WBCs of PEG-GCSF and GCSF-Fc treated groups were significantly higher than the corresponding values of GCSF treated group (Fig. 5C and D). The activity of GCSF-Fc compared to GCSF started to diminish on day 7 when the numbers of neutrophil and WBC in GCSF-Fc and GCSF groups equilibrated. The level of WBCs of the GCSF group was similar while the neutrophil levels were relatively higher than that of the PBS group. According to these data, both PEG-GCSF and GCSF-Fc were effective for resolving neutropenia with a single injection, but GCSF-Fc was less effective compared to PEG-GCSF (Fig. 5). Importantly, there was no obvious change in the number of red blood cells and platelets during the course of the experiments (data not shown).

**Figure 6:**
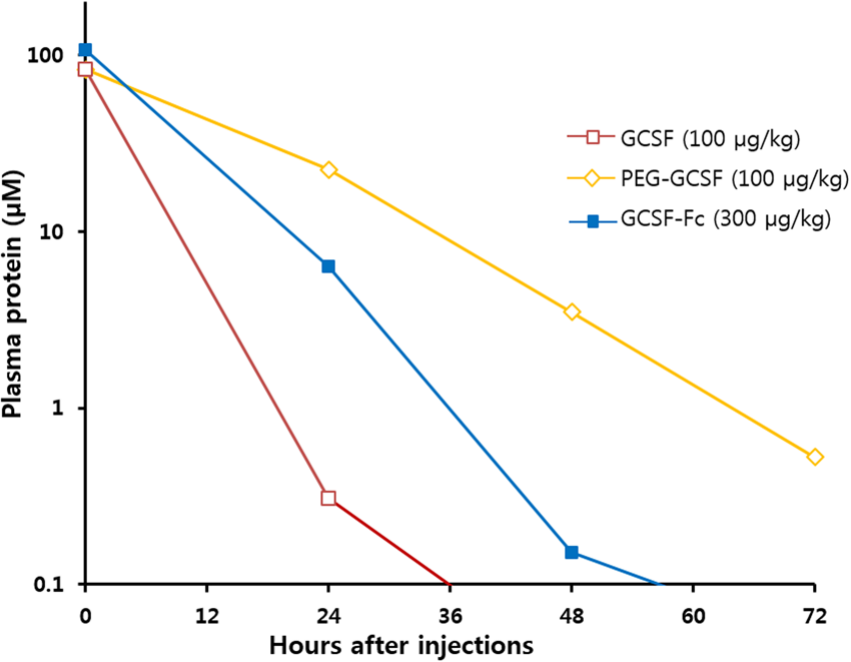
Protein level in plasma after administration of GCSF-Fc, PEG-GCSF and GCSF. The measurements were performed at 24 h, 48 h and 72 h after protein injection. Three groups of rats were utilized for plasma protein measurement, GCSF (100 µg/kg), PEG-GCSF (100 µg/kg) and GCSF-Fc (300 µg/kg). Data are given as means of ± SE of 3 rats/group. The data points at Day 0 were the amount of supplementary proteins, assuming that all proteins were diluted completely in rats’ body plasma.

Twenty-four, 48 and 72 hours after protein injection, the duration of proteins in the plasma was measured using a GCSF ELISA kit. Figure 6 shows that the elimination of GCSF was more rapid than GCSF-Fc and PEG-GCSF. Twenty-four hours after injection, GCSF was completely cleared compared to more than 48 h of GCSF-Fc and more than 72 h of PEG-GCSF. These data show that conjugation with PEG or fusion with Fc domain can significantly increase circulating levels of GCSF due to a prolonged half-life.

## Discussion

In this study, we described in detail the soluble prokaryotic expression and simple purification of GCSF-Fc by fusion technology with an MBP tag and conventional chromatography. We demonstrate that IgG1 Fc and PEG do not appreciably affect GCSF activity as evidenced by the similarity in *in vitro* bioactivity. Our *in vivo* experiments in neutropenic rats revealed that PEG-GCSF was more effective than GCSF-Fc in neutrophil and WBC recovery as well as stability of the protein in plasma.

Recombinant proteins commonly form inclusion bodies when expressed in the cytoplasm of *E. coli*. Fusion technology with tags such as MBP, N-utilization substance protein A, or protein disulfide bond isomerase has been shown to be effective for enhancing protein expression as well as solubility^16, 19^. Because of its relatively small size, easy purification using MBP chromatography, and efficacy in supporting the expression as well as proper folding of target proteins in the reducing environment of *E. coli* cytoplasm, MBP is largely the tag of choice. For the expression of MBP-tagged GCSF-Fc, induction by 0.5 mM IPTG at 30°C for 5 h was the optimal condition for expression (Fig. 1B). Nearly 70% of this fusion protein was soluble and this maximized the purification process with only the protein A resin (Fig. 2A and B). When the Fc domain alone was expressed in *E. coli* BL21(DE3), multimer and soluble aggregates were formed (data not shown), which may be due to poor disulfide bond formation. To overcome this, MBP-tagged GCSF-Fc was expressed in the Shuffle T7 Express system to support disulfide bond formation. Shuffle T7 Express is an engineered *E. coli* B strain that creates an oxidizing cytoplasm environment by deleting genes of glutaredoxin reductase and thioredoxin reductase combined with a mutation in the peroxiredoxin enzyme. Additionally, this strain continuously expresses the disulfide bond isomerase DsbC in the cytoplasm to promote the correction of mis-oxidized proteins into their correct form^20^. Under these defined conditions, the homodimer of GCSF-Fc protein was successfully purified (Fig. 2D).

PEGylation has several advantages for *in vitro* protein engineering including: protection the target protein by increasing stability, reducing renal clearance, and mitigating toxicity^21^. To date, numerous strategies for GCSF PEGylation have been applied, including conjugating PEG at the N-terminus, coupling PEG at cysteine 17 or lysine 41, and PEGylation in organic solvent^18, 22–24^. Covalent attachment of 20 kDa PEG at the N-terminus residue of GCSF by reductive alkyl conjugation using mPEG-CHO showed the best conjugation efficacy while remaining biologically active^18, 25^. These previous studies performed the reaction at pH 5^18, 25^ therefore, we also lowered the pH to 5 and found that the purified GCSF was completely aggregated (data not shown). The calculated pI of GCSF was 5.65 and when we reduced pH from 8 to 5, the protein began to aggregate at pH of 5.65. Therefore, we performed the conjugation at pH 6 that allowed the PEGylation reaction to proceed. However, the efficiency was low compared to the high PEGylation yield of previous reports which reached 90% or higher^18, 25^. Our GCSF was obtained from MBP-GCSF using TEV protease for tag removal, resulting in a remaining glycine residue at the N-terminus (Gly-GCSF). To test whether the low PEGylation efficacy was caused by this N-terminal glycine, we purified non-Gly GCSF from PDIb´a´-GCSF fusion construct using EKL protease (Supplementary Fig. S5). The conjugation efficiency of this non-Gly GCSF was more than 90% (Supplementary Fig. S6), indicating that the remaining glycine from TEV protease cleavage at the N-terminus significantly reduced the conjugation efficiency of N-terminal, site-specific, mono-PEGylation of GCSF.

Upon cation exchange chromatography, the PEG-GCSF was eluted earlier than the unreacted GCSF, while neutral unreacted PEG did not bind to the column (Fig. 3C). This may be by cause of the PEG shields the surface charges of GCSF^21^. On SDS-PAGE, PEG-GCSF with high purity appeared at size of approximately 50 kDa which was higher than the reported molecular weight (Fig. 3B), and is likely caused by PEGs capacity to occupy a larger volume in an aqueous environment^25^.

*In vitro* proliferation experiment demonstrated that the conjugation of PEG at the N-terminus of GCSF did not negatively impact its biological activity (Fig. 4). However, others have reported a decrease of *in vitro* bioactivity when the protein was conjugated to PEG^26–28^. GCSF-Fc showed lower *in vitro* proliferation activity compared to GCSF, which is consistent with a previous report^8^.

According to previous pharmacokinetics experiments, GCSF-Fc produced in mammalian cells has 5- to 8- times longer half-life than GCSF^8, 9^. Despite this advantage, the eukaryotic production process can be both complex and time consuming. Our study was designed to produce the therapeutic protein fused with the IgG1 Fc region in *E. coli* and to test the half-life and the effectiveness *in vitro* and *in vivo*. Our results show that GCSF-Fc can confer added benefits (e.g. stability) compared to native GCSF. While GCSF-Fc was more effective than GCSF, it was less effective than PEG-GCSF in our *in vivo* experiments. Our results are disparate from a previous report^9^ that showed GCSF-Fc had similar activity to PEG-GCSF *in vivo*. The lessened effect of GCSF-Fc produced from *E. coli* may be due to the reduced stability of non-glycosylated Fc domain produced in *E. coli* compared to glycosylated Fc domain^29, 30^. Or, the non-glycosylated Fc from *E. coli* might have lower affinity to the FcRn. In general, the fusion of GCSF-Fc was produced effectively in *E. coli* and it had an improved circulating half-life and hematopoietic properties compared to GCSF. Our study strongly suggests that, in order to be developed as a biodrug produced from a prokaryote in the future, the GCSF-Fc, especially the Fc domain, must be mutated or modified for higher stability or higher affinity to FcRn. Collectively, these data are important for cellular engineering of bioactive GCSF.

## Electronic supplementary material

Supplementary Information

## References

[1] Root RK, Dale DC (1999). Granulocyte colony-stimulating factor and granulocyte-macrophage colony-stimulating factor: comparisons and potential for use in the treatment of infections in nonneutropenic patients. The Journal of infectious diseases.

[2] Demetri GD, Griffin JD (1991). Granulocyte colony-stimulating factor and its receptor. Blood.

[3] Almenar D (2009). Pegfilgrastim and daily granulocyte colony-stimulating factor: patterns of use and neutropenia-related outcomes in cancer patients in Spain–results of the LEARN Study. European journal of cancer care.

[4] Yang BB, Kido A (2011). Pharmacokinetics and pharmacodynamics of pegfilgrastim. Clinical pharmacokinetics.

[5] Molineux G (2004). The design and development of pegfilgrastim (PEG-rmetHuG-CSF, Neulasta). Current pharmaceutical design.

[6] Halpern W (2002). Albugranin, a recombinant human granulocyte colony stimulating factor (G-CSF) genetically fused to recombinant human albumin induces prolonged myelopoietic effects in mice and monkeys. Pharmaceutical research.

[7] Zhao S (2013). Extending the serum half-life of G-CSF via fusion with the domain III of human serum albumin. BioMed research international.

[8] Cox GN (2004). Enhanced circulating half-life and hematopoietic properties of a human granulocyte colony-stimulating factor/immunoglobulin fusion protein. Experimental hematology.

[9] Cox GN (2014). Hematopoietic properties of granulocyte colony-stimulating factor/immunoglobulin (G-CSF/IgG-Fc) fusion proteins in normal and neutropenic rodents. PloS one.

[10] Harris, J. M. In *Poly(Ethylene Glycol) Chemistry, Biotechnical and Biomediacal Applications* (ed J. Milton Harris) Ch. 1, (Springer US, 1992).

[11] Palumbo E (2009). PEG-interferon in acute and chronic hepatitis C: a review. American journal of therapeutics.

[12] Veldhuis JD, Bidlingmaier M, Bailey J, Erickson D, Sandroni P (2010). A pegylated growth hormone receptor antagonist, pegvisomant, does not enter the brain in humans. The Journal of clinical endocrinology and metabolism.

[13] Wang B, Cao Y, Chi S, Lou D (2012). A PEGylation technology of L-asparaginase with monomethoxy polyethylene glycol-propionaldehyde. Zeitschrift fur Naturforschung. C, Journal of biosciences.

[14] Ghetie V, Ward ES (2002). Transcytosis and catabolism of antibody. Immunologic research.

[15] Scott LJ (2014). Etanercept: a review of its use in autoimmune inflammatory diseases. Drugs.

[16] Do BH, Ryu HB, Hoang P, Koo BK, Choe H (2014). Soluble prokaryotic overexpression and purification of bioactive human granulocyte colony-stimulating factor by maltose binding protein and protein disulfide isomerase. PloS one.

[17] Busso D, Delagoutte-Busso B, Moras D (2005). Construction of a set Gateway-based destination vectors for high-throughput cloning and expression screening in Escherichia coli. Analytical biochemistry.

[18] Kinstler OB (1996). Characterization and stability of N-terminally PEGylated rhG-CSF. Pharmaceutical research.

[19] Vu TT (2015). Soluble overexpression and purification of bioactive human CCL2 in E. coli by maltose-binding protein. Molecular biology reports.

[20] Lobstein J (2012). SHuffle, a novel Escherichia coli protein expression strain capable of correctly folding disulfide bonded proteins in its cytoplasm. Microbial cell factories.

[21] Veronese, F. M. In *PEGylated Protein Drugs: Basic Science and* Clinical *Applications* (ed Francesco M. Veronese) 147–164 (Birkhäuser Basel, 2009).

[22] Veronese FM (2007). Site-specific pegylation of G-CSF by reversible denaturation. Bioconjugate chemistry.

[23] Peng F (2014). PEGylation of G-CSF in organic solvent markedly increase the efficacy and reactivity through protein unfolding, hydrolysis inhibition and solvent effect. Journal of biotechnology.

[24] Mero A (2016). Site-selective enzymatic chemistry for polymer conjugation to protein lysine residues: PEGylation of G-CSF at lysine-41. Polym Chem-Uk.

[25] Kinstler O, Molineux G, Treuheit M, Ladd D, Gegg C (2002). Mono-N-terminal poly(ethylene glycol)-protein conjugates. Advanced drug delivery reviews.

[26] Tanaka H, Satake-Ishikawa R, Ishikawa M, Matsuki S, Asano K (1991). Pharmacokinetics of recombinant human granulocyte colony-stimulating factor conjugated to polyethylene glycol in rats. Cancer research.

[27] Bowen S (1999). Relationship between molecular mass and duration of activity of polyethylene glycol conjugated granulocyte colony-stimulating factor mutein. Experimental hematology.

[28] Gaertner HF, Offord RE (1996). Site-specific attachment of functionalized poly(ethylene glycol) to the amino terminus of proteins. Bioconjugate chemistry.

[29] Zheng K, Bantog C, Bayer R (2011). The impact of glycosylation on monoclonal antibody conformation and stability. mAbs.

[30] Reusch D, Tejada ML (2015). Fc glycans of therapeutic antibodies as critical quality attributes. Glycobiology.

