# Supplementary material for: Granulocyte colony-stimulating factor (GCSF) fused with Fc Domain produced from E. coli is less effective than Polyethylene Glycol-conjugated GCSF
Source: Sci Rep. 2017 Jul 25;7:6480. doi: 10.1038/s41598-017-06726-7 (PMC5526978; doi:10.1038/s41598-017-06726-7)
Supplement: Supplementary file 1 — Supplementary Information [file 41598_2017_6726_MOESM1_ESM.doc]

**Granulocyte Colony-Stimulating Factor (GCSF) fused with Fc Domain produced from *E. coli* is less effective than Polyethylene Glycol-conjugated GCSF.**

Bich Hang Do^1^, Hyo Jeong Kang^1^, Jung-A Song^1^, Minh Tan Nguyen^1^, Sangsu Park^1^, Jiwon Yoo^1^, Anh Ngoc Nguyen^1^, Grace G. Kwon^1^, Jaepyeong Jang^1^, Mihee Jang^1^, Sunju Lee^1^, Seoungjun So^1^, Seongrak Sim^1^, Kyung Jin Lee^2^, Mark J Osborn^1,3^, and Han Choe^1^,*

**Supplementary Figures**


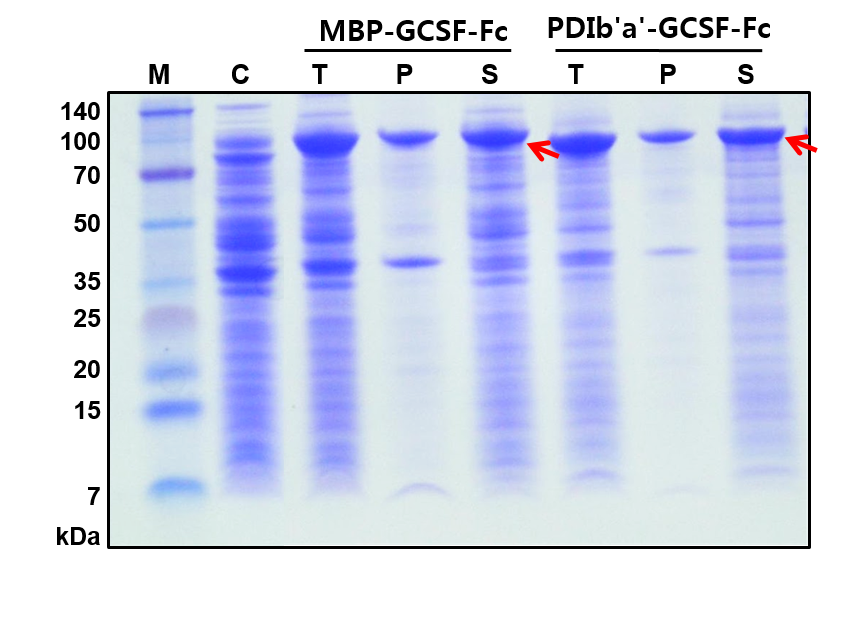


#### Figure S1. Expression of MBP tagged and PDIb'a' tagged GCSF-Fc expressed in *E. coli* BL21(DE3). M, molecular weight marker; C, total cell protein before IPTG induction; I, total cell protein after IPTG induction; P, pellet fraction after sonication; S, soluble fraction after sonication.

####
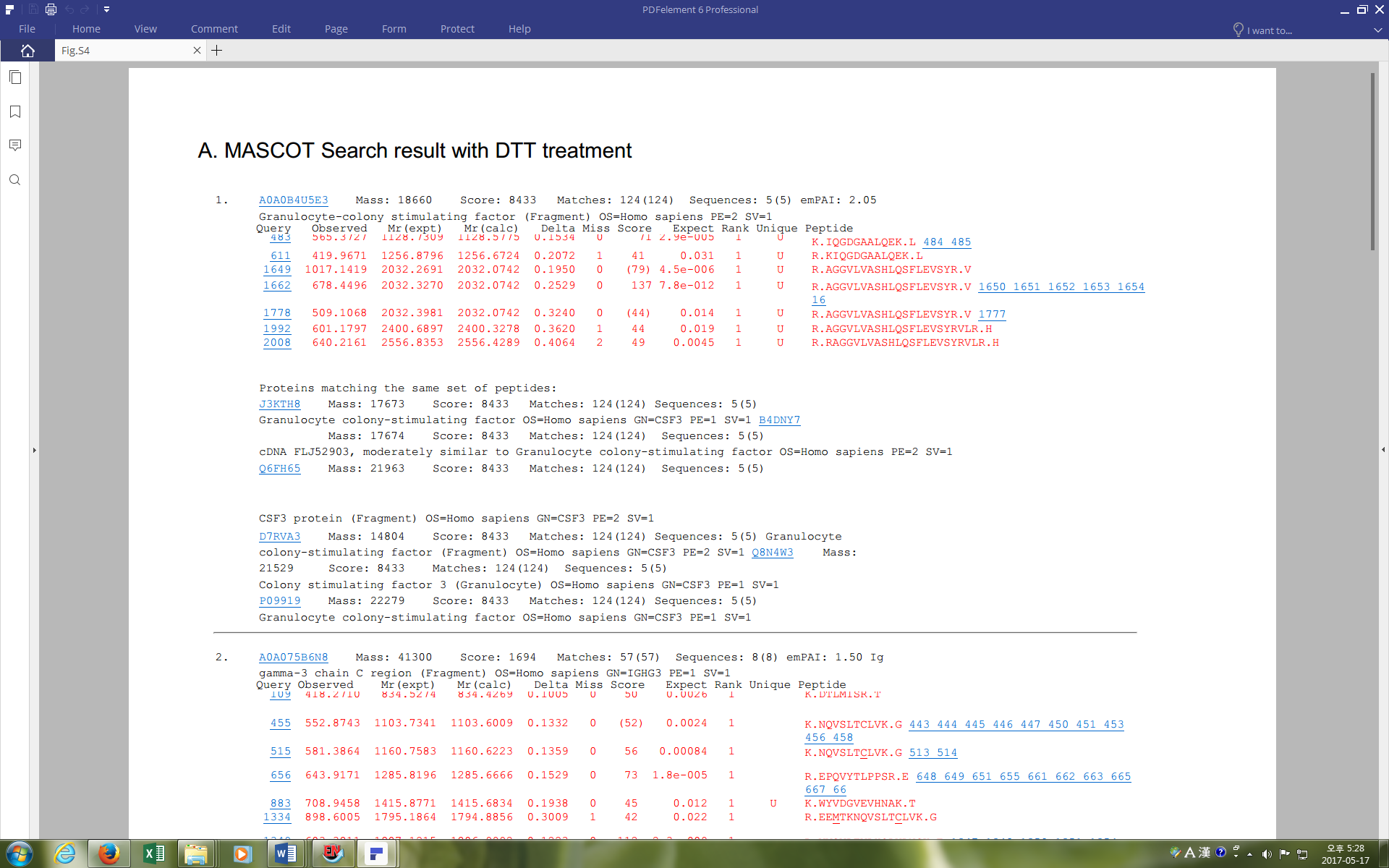


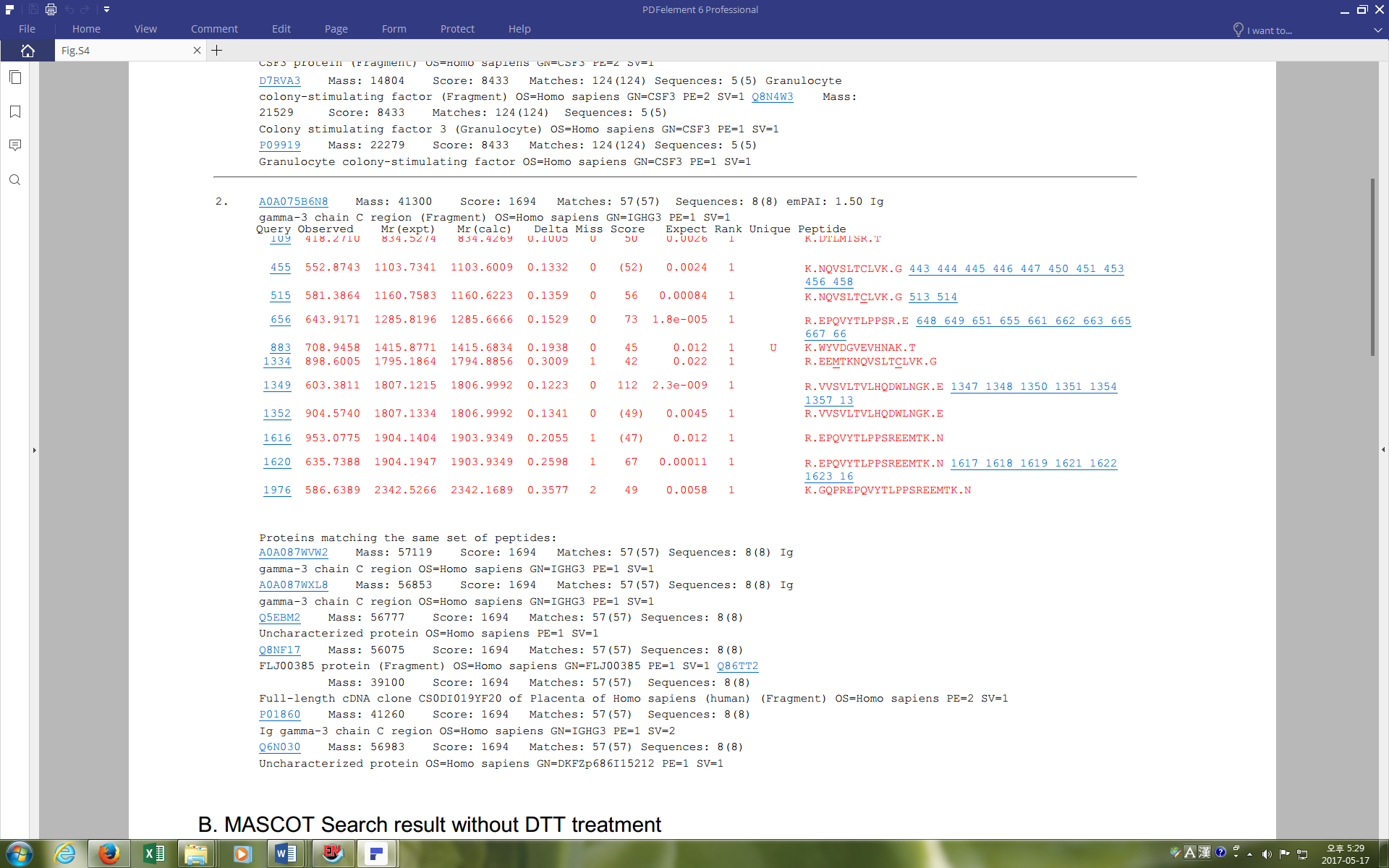


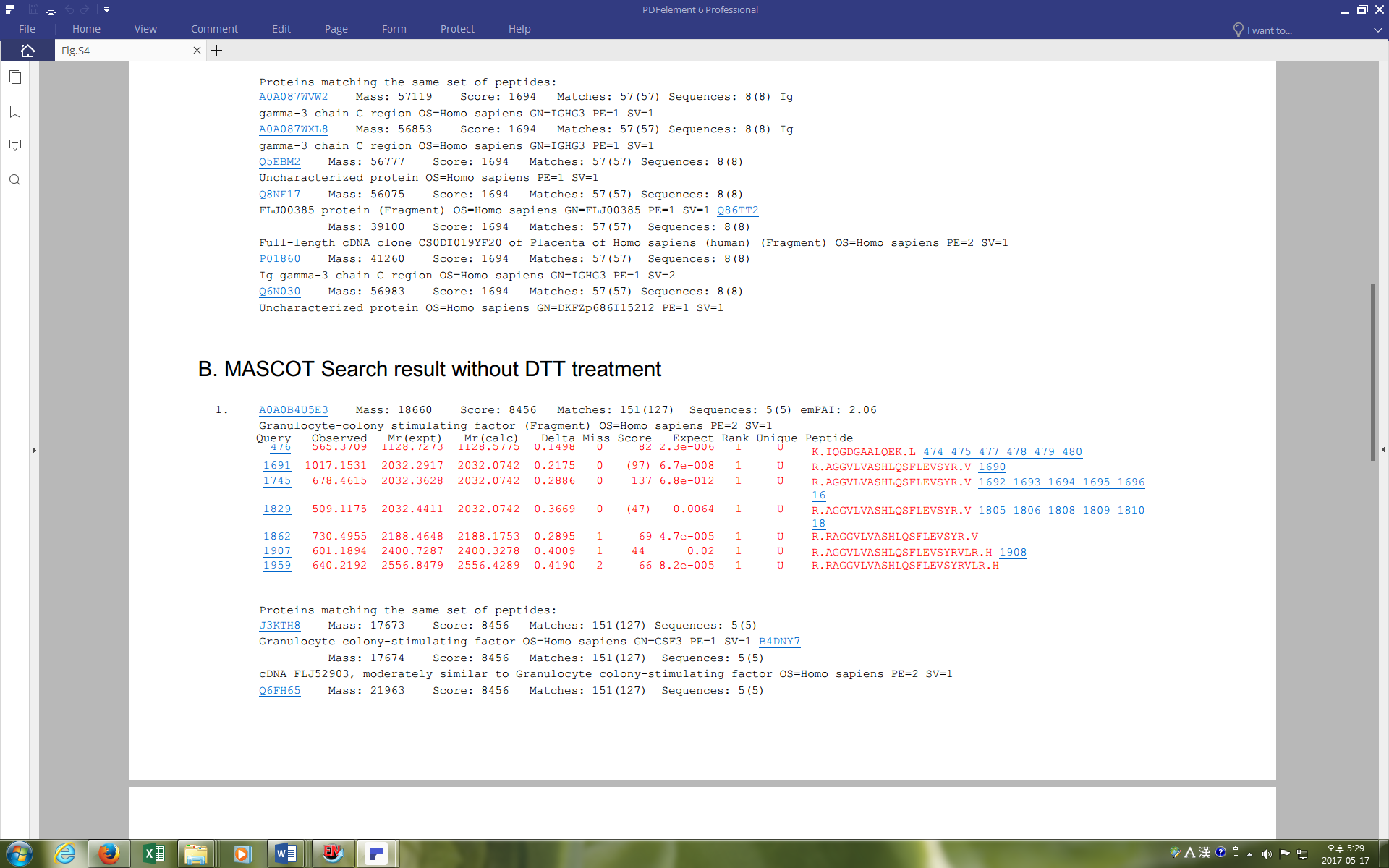


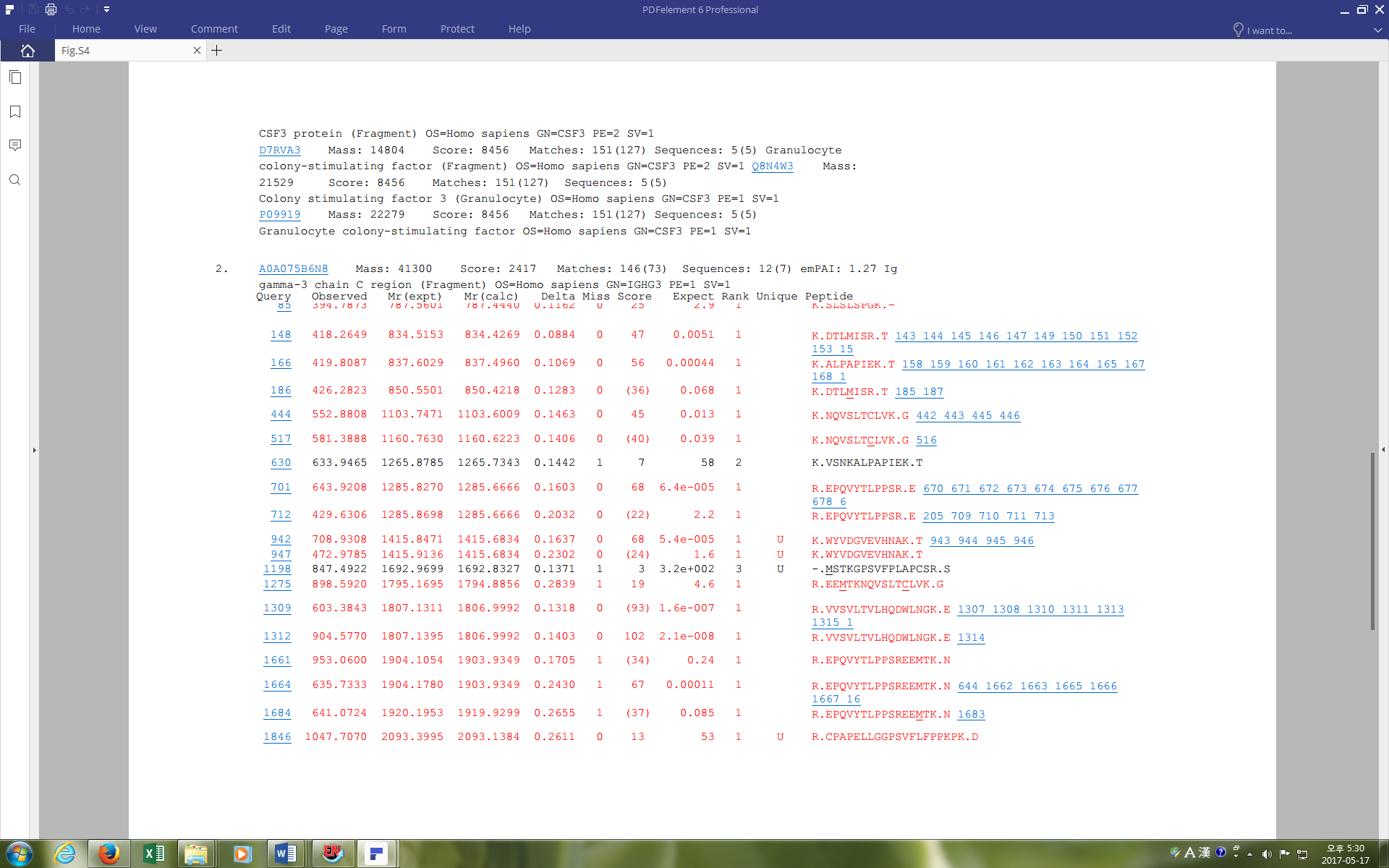


#### Figure S2. The result of Mascot search of LC-MS/MS data under reducing (A) and non-reducing (B) conditions.


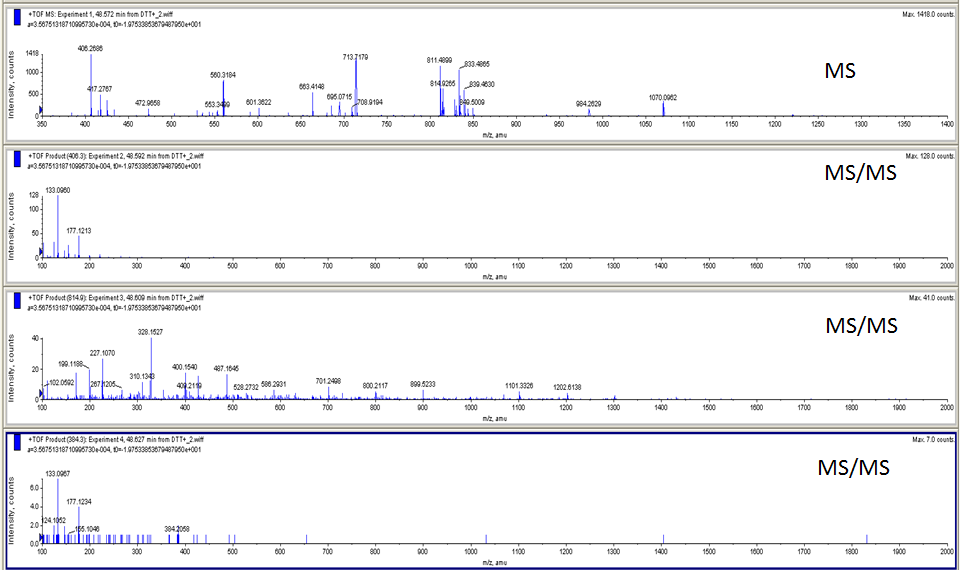


**Figure S3. Mass analysis of purified GCSF-Fc under reducing (with DTT) condition**.

The GCSF-Fc was reduced with 10mM DTT before applying onto LC-MS/MS. MS spectra were collected in full scan mode (350–1400 Da) followed by three MS/MS scans of the most intense ions.


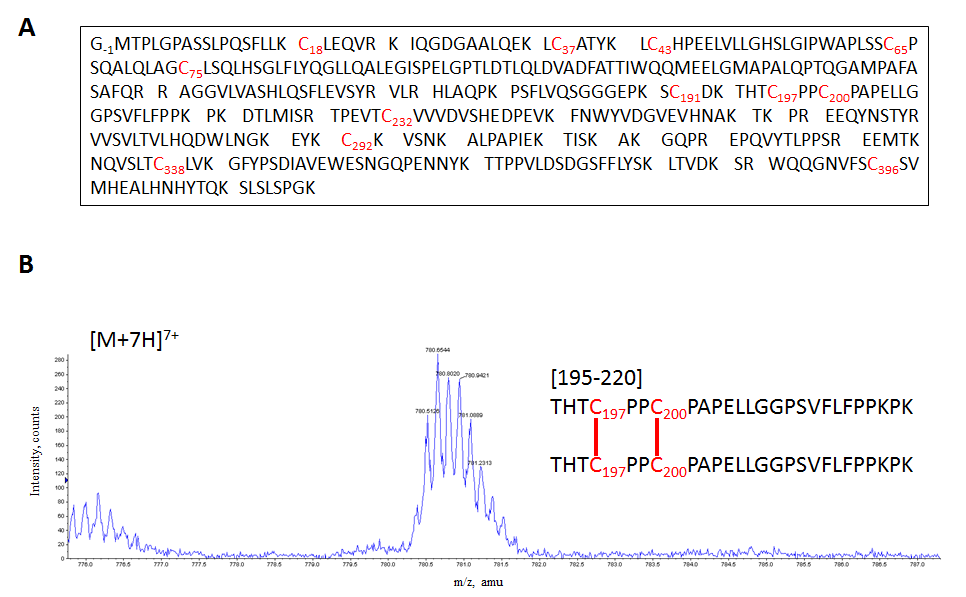


**Figure S4. Mass analysis of the disulfide-linked peptide under non-reducing condition.** (A) Tryptic peptide map of GCSF-Fc. (B) Annotated MS/MS spectrum of the disulfide-linked peptide (fragment of 95-220 amino acids) under non-reducing condition observed at *m/z* value 780.51^7+^ containing 2 inter-disulfide bonds in Fc region (C_197_-C_197_ and C_200_-C_200_).


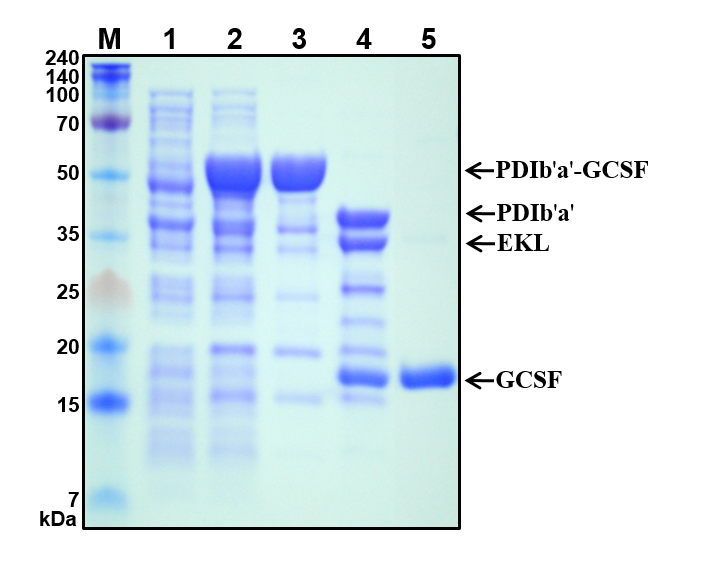


#### Figure S5. Purification of non-Gly GCSF from PDIb'a'-GCSF fusion protein using EKL protease for tag removal. SDS-PAGE analysis of GCSF purification process. M, molecular weight marker; lane 1, total cell extract before IPTG induction; lane 2, soluble fraction after IPTG induction and cell homogenization; lane 3, PDIb'a'-GCSF fusion protein was purified by HisTrap HP column; lane 4, PDIb'a'-GCSF fusion protein was cleaved by EKL protease; lane 5, final GCSF after purifying by HisTrap HP column. The arrows indicate positions of fusion PDIb'a'-GCSF (59.1 kDa) and GCSF (18.8 kDa).


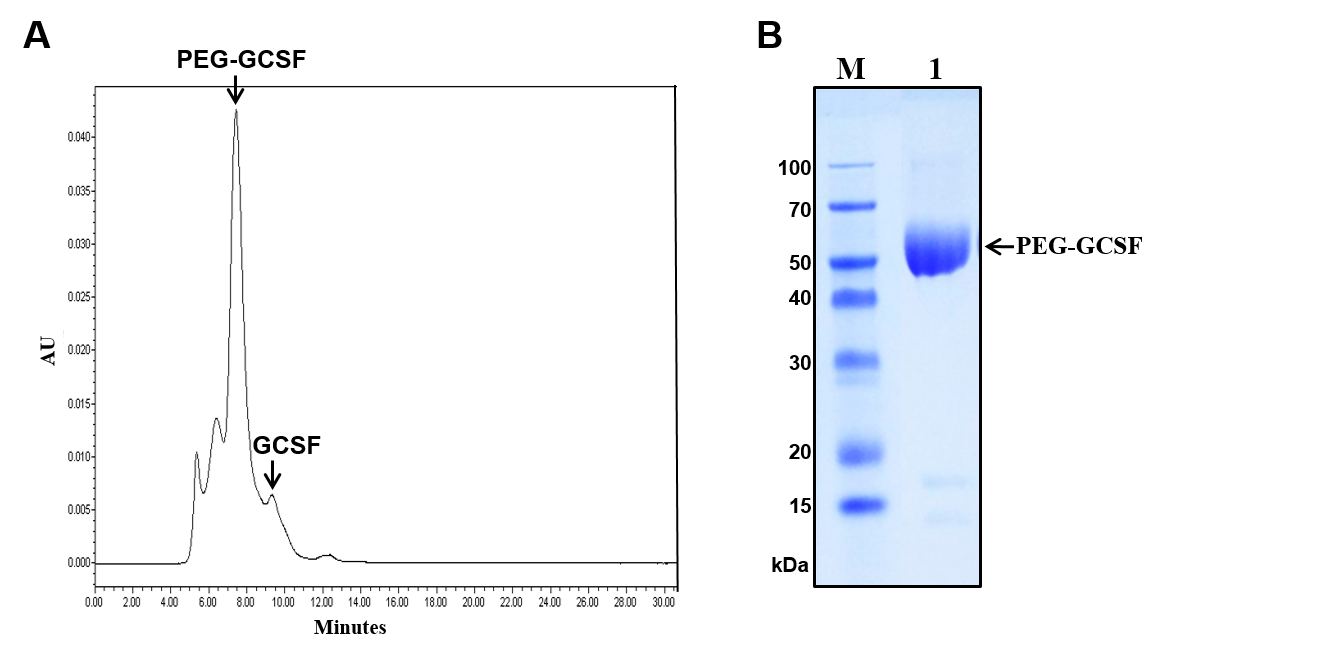


#### Figure S6. PEGylation of non-Gly GCSF. (A) SEC-HPLC analysis of reaction mixture for checking PEGylation efficiency. The arrows indicate the peak of PEG-GCSF eluted at 7.44 min and peak of GCSF eluted at 9.32 min. (B) SDS-PAGE analysis of PEG-GCSF collected from PEG-GCSF peak.
